# Matrix metalloproteinase-8 levels in oral samples as a biomarker for periodontitis in the Chinese population: an observational study

**DOI:** 10.1186/s12903-018-0512-8

**Published:** 2018-03-27

**Authors:** Chao Yuan, Xiaochen Liu, Shuguo Zheng

**Affiliations:** Department of Preventive Dentistry, National Engineering Laboratory for Digital and Material Technology of Stomatology, Beijing Key Laboratory of Digital Stomatology, Peking University School and Hospital of Stomatology, 22 Zhongguancun Avenue South, Haidian District, Beijing, 100081 People’s Republic of China

**Keywords:** Matrix metalloproteinase-8, Diagnostic biomarker, Periodontitis

## Abstract

**Background:**

Clinical evaluation of periodontal inflammation does not fully reflect the disease activity. Extensive studies have been conducted out on gingival crevicular fluid (GCF) components that might serve as potential diagnostic markers for periodontitis, among which matrix metalloproteinase-8 (MMP-8) has shown to be promising, but there were no studies for individuals in China. The aim of this study was to compare clinical diagnostic parameters and levels of active MMP-8 (aMMP-8) in GCF and oral rinse samples from the Chinese patients with varying degrees of periodontal inflammation.

**Methods:**

GCF and oral rinse samples were obtained from 60 participants into two groups, a periodontitis group and a control group, specified by the presence and number of pocket depths or attachment loss. The aMMP-8 levels in GCF and oral rinse samples was quantified by ELISA using specific monoclonal antibodies. Logistic and linear regression models were employed for testing the correlation between aMMP-8 levels and periodontal condition, as well as diagnostic sensitivity and specificity.

**Results:**

Periodontitis group (mean = 24.84 ng/ml) exhibited significantly higher aMMP-8 levels than control group in GCF (*p* < 0.001). The aMMP-8 levels in oral rinse samples ranged from 0.05 to 2.18 ng/ml, but differences were not statistically significant between the two groups (*p* > 0.1). Receiver operating characteristic curve analysis showed a highest threshold of 6.66, with a corresponding sensitivity and specificity of 0.8 and 0.9, respectively.

**Conclusions:**

Measuring aMMP-8 levels in GCF may have potentiality for complementary early diagnosis of periodontal disease and inflammation in the Chinese population.

## Background

Periodontitis is a general chronic inflammatory condition that is initiated by subgingival colonisation by periodontopathogenic bacteria, causing an inflammatory and destructive host response in susceptible patients [1–3]. Diagnosis of periodontal disease is traditionally based on clinical parameters and indices that reflect a history of periodontal diseases but cannot fully reflect disease activity. Early diagnosis of ongoing tissue destruction in periodontitis is particularly important in order to prevent further attachment loss. Unlike other areas of medicine, biochemical testing is not regularly used in dentistry, despite obvious advantages [4] for supplementing or replacing traditional diagnostic methods.

Infection of periodontal tissues by pathogenic microorganisms can trigger mechanisms of inflammatory defence that produce cytokines and enzymes prominent among which are the matrix metalloproteinases (MMPs), leading to tissue degradation and bone resorption [3, 5]. Among the MMPs family, the active form of MMP-8 (aMMP-8) in GCF [6] is the most prominent collagenase (collagenase 2) associated with periodontitis [7, 8]. Measuring single-site GCF aMMP-8 levels could enable the clinician to distinguish between healthy tissues and those with gingivitis or periodontitis [9–11].

Oral rinse samples consist more of GCF from periodontal pockets than saliva [12–14], which contains sloughed epithelial cells from oral mucous membranes, nasopharyngeal discharge, food debris, and bacteria and their metabolic products. Thus, analysis of oral rinse may be useful in the monitoring of general periodontal disease status, and facilitate early identification of individuals at risk of periodontitis [13].

The aim of this study is to investigate the relationship between clinical diagnostic parameters and the point-of-care testing levels of aMMP-8 in GCF and oral rinse in individuals with periodontal disease in China, and to test if the aMMP-8 levels could be effective for monitoring the status of periodontal disease.

## Methods

### Patients, clinical assessments and samples

A total of 60 Chinese participants with/without periodontitis aged mean (95% CI) of 36.0 (16.4–55.6) years were enrolled for an observational study from the Department of Preventive Dentistry, Peking University School and Hospital of Stomatology, Beijing, China. All individuals had signed informed consent to participate in this study, and they all had at least 24 teeth but various periodontal health status. The classification of periodontal status was modified based on the criteria of Classification of Periodontal Diseases and Conditions by American Academy of Periodontology [15]. Patients were categorized into two groups according to their clinical periodontal condition, determined by combining the pocket probing depth (PD) and attachment loss (AL). The groups were as follows: (1) Control group – subjects without any loss of attachment interdentally of greater than 2 mm. (2) Periodontitis group – subjects with a diagnosis of moderate / severe chronic periodontitis using standard AAP / CDC case definitions of periodontal disease, namely at least two teeth with 4 mm or more interproximal periodontal attachment loss or two teeth with at least 5 mm interproximal pocket depths. According to the exclusion criteria, all study participants were non-smokers, had no history of systemic diseases, and underwent no treatment with medication during the last 12 weeks before examination and sampling. Individuals were excluded if they had untreated caries, wore orthodontic appliances, or were pregnant or currently at the period of breast-feeding. Individuals that had previously undergone periodontal therapy within 1 year before the start of the study were also excluded. Patients who fulfilled all the above criteria were included, and the study protocol was approved by the Local Ethics Committee at the Peking University School and Hospital of Stomatology (No. PKUSSIRB-2012055).

All parameters were assessed and aMMP-8 levels were recorded by one single examiner. GCF samples were obtained from mesio-buccal sites on the index teeth at 16, 11, 26, 36, 31 and 46. After application of cotton rolls and gentle air-drying, specific dentoELISA strips(Dentognositcs GmbH, Germany) were placed at the margin of the crevice and inserted carefully into the gingival sulcus to a depth of less than 2 mm to avoid soft tissue damage. Strips were left in place for 30s, and then transferred to the tubes for sample collection. Sample strips contaminated with blood were discarded, and the sampling procedure for these participants would be repeated after 30 min. Pipette 600 μl of sample washing buffer from dentoELISA kit was added to the sample in the transportation tubes containing GCF strip. After removal of GCF strip, the eluted sample was frozen and stored at − 80°C till needed [16].

### Oral rinse samples

To collect oral rinse samples, 5 ml of tap water was placed into the oral cavity using a disposable plastic pipette, after which all participants rinsed their mouth for 30s, and the rinse was transferred to the tubes for sample collection as previously described [17]. On the same day, an aliquot of the rinse sample was used for measurement of aMMP-8 activity, and the remainder was frozen and stored at − 80°C till needed.

### Determination of aMMP-8 levels

The dentoELISA is an Enzyme immunoassay for the quantification of aMMP-8 in GCF. It could automatically conduct the whole assay process, that is, steps like liquid handling as well as readout based on a software program and a robust algorithm. The key component is a cartridge consisting of a liquid-handling module containing all relevant reagents for immunological reactions like clinical sample, conjugate, wash buffers, and substrate and a reaction chamber containing six filters including positive and negative controls, where the immunological reactions take place [18, 19].

After elution from the strips, the aMMP-8 levels was measured by ELISA using monoclonal antibodies 8708 and 8706 (DentalELISA, Germany) according to established procedures [18, 19]: The GCF eluate should be diluted 1:50 before testing, then 100 μl of the diluted sample was used to measure the absorbance of each well at 450 nm with background reference at 620 nm.

Clinical parameters including PD, bleeding on probing (BOP) and Bleeding Index (BI) [20] were recorded using GCF samples obtained from the same sites on the index teeth.

### Data analysis

Blind statistical analysis was performed by a third-party professional statistician. The authors of this article hadn’t access to information that could identify individual participants during or after data collection. Descriptive statistics of the social background, periodontal clinical measures and aMMP-8 levels for all individuals were performed. Independent t-tests were performed to determine statistical significance between the study groups. Logistic regression models were used to testing correlations between aMMP-8 levels and periodontal condition.

The association of nominal variables in cross tables was tested using the Cramer V test, and the diagnostic sensitivity and specificity of MMP-8 measuring methods were evaluated by receiver operating characteristic curve analysis. *P* < 0.05 were considered statistically significant, and statistical analyses were performed using SPSS 17.0 software (IBM, United States) [21].

## Results

### Clinical examination and biomarker test

GCF samples were obtained from 358 sites on the index teeth of 60 participants with an average age of 35.8 years. Parameters from Group 2 (shown in Table 1) individuals (mean PD [95% CI]: 5.42 [2.05–8.79]; mean BI [95% CI]: 3.43 [1.45–5.41], BOP: 95.1%) were significantly higher than Group 1 (*P* < 0.01).

**Table 1:** Clinical parameters of 358 index tooth sites from 60 participants

		PD		BI		BOP
Group	Sites	Mean	95% CI	Mean	95% CI	%
1	180	2.48	1.36, 3.60	1.41	−0.67, 3.49	42.5
2	178	5.42	2.05, 8.79	3.43	1.45, 5.41	95.1
Total	358	3.74	0.02, 7.46	2.28	−0.54, 5.10	73.9

*PD* Probing depth, *BI* Bleeding index, *BOP* Bleeding on probing

Among all the 358 GCF samples (Table 2), the aMMP-8 levels ranged from 1.34–121.43 ng/ml, with significantly higher level (*P* < 0.001) coming up in Group 2 (mean value [95% CI]: 24.84 [(− 14.11)-63.79] ng/ml). Among all the 60 oral rinse samples (Table 3), the aMMP-8 levels range was 0.05–2.18 ng/ml, and differences between groups were not significant (*P* > 0.1). The estimated Pearson’s correlation coefficient between the average of the aMMP-8 level obtained in CGF and that in oral rinse was 0.14 with *P* = 0.932 based on the test of significance. This indicates no significant statistical association between the two biological fluids.

**Table 2:** Levels of aMMP-8 in GCF samples from 358 index tooth sites from 60 participants (ng/ml)

Group	Sites	Mean	95% CI	Min	Max	Med
1	180	4.88	−3.78, 13.54	1.34	44.54	4.08
2	178	24.84	−14.11, 63.79	5.34	121.43	18.54
Total	358	13.44	−19.19, 46.07	1.34	121.43	6.32

Group 1 and 2, Independent samples T test, *p* < 0.001

**Table 3:** Levels of aMMP-8 in oral rinse samples from 60 participants (ng/ml)

Group	N	Mean	95% CI	Min	Max	Med
1	30	0.77	−0.07, 1.61	0.05	2.18	0.73
2	30	0.59	−0.15, 1.35	0.21	1.36	0.46
Total	60	0.68	−0.12, 1.48	0.05	2.18	0.64

Group 1 and 2, Independent samples T test, *p* = 0.158

### Periodontitis and levels of aMMP-8 logistic regression model

Model fitting (Table 4) showed that samples containing a high levels of aMMP-8 were more likely to source from individuals with periodontitis (*P* = 0.24).

**Table 4:** Logistic regression model of periodontitis and aMMP-8 levels

	Estimate	S.E.	z value	Pr (>|z|)
	−2.19	0.30	−7.19	< 0.01
Levels	0.24	0.04	6.61	< 0.01

Receiver operating characteristic curve analysis was subsequently performed. Calculation of the area under the curve showed a highest threshold of 6.66, and a corresponding sensitivity and specificity of 0.8 and 0.9, respectively.

### Association between aMMP-8 and PD/BI

A linear regression model was fitted with PD/BI treated as the outcome and aMMP-8 as the diagnosis biomarker (Table 5). The model showed a strong correlation between aMMP-8 levels and PD/BI, with an increase in aMMP-8 of 16.67 (1/0.06) correlating with an increase in PD of 1, and with an increase in aMMP-8 of 20 (1/0.05) correlating with an increase in BI of 1.

**Table 5:** Logistic regression model of PD/BI and aMMP-8 levels

	Estimate	S.E.	t value	Pr (>|t|)
aMMP-8~PD	0.06	0.01	10.42	0.00
aMMP-8~BI	0.05	0.00	10.74	< 0.01

## Discussion

At present, regular measurement of attachment loss is the best choice for monitoring chronic periodontitis in the periodontal pocket [22]. However, monitoring AL is not suitable for early detection and poor at establishing disease prognosis. Indeed, probing depth, attachment loss and X-ray analysis only reflect historical damage to the periodontal pocket, and are thus not helpful for monitoring [23]. During the stable periods of periodontitis, the levels of aMMP-8 remained low, but the damage to the periodontal pocket and attachment loss could persist.

Similarly, the primary value of BOP and BI as a diagnostic sign is that its presence indicates that the tissues are inflamed, but it is likely that the amount of probing pressure can influence whether a site bleeds or not. Perhaps only tissue slices are able to reflect the actual pathological state, but histological diagnosis is a much difficult, expensive and time-consuming way of diagnosis.

In contrast, biomarkers serve as a convenient method for distinguishing between healthy and diseased tissues, or for learning about the response to pharmacological or other therapeutic interventions [24]. In fact, biomarkers are potentially useful throughout the entire disease process, as they can be used at the periods of: (1) before diagnosis, for screening and risk assessment; (2) during diagnosis, for determining staging, grading and selection of appropriate therapies; (3) after diagnosis, for monitoring therapeutic outcomes and disease remission.

Our results demonstrate a clear positive correlation between aMMP-8 levels and clinical periodontal index, including PD or BI parameters, suggesting it could be used as a biomarker for diagnosis. In line with previous studies [25], receiver operating characteristic curve analysis showed that the highest aMMP-8 threshold was 6.66, with corresponding sensitivity and specificity values of 0.8 and 0.9, respectively. These values represent a significant improvement beyond existing clinical indicators that are unable to quantify the activity of periodontitis, and facilitate early detection and diagnosis prior to the appearance of inflammatory damage to periodontal tissue. However, more thorough investigation of threshold values through longitudinal studies with larger sample size are still needed to push this in further.

Recent proteomic analyses suggested that the whole saliva contains more than 3000 different proteins belonging to various classes and that are involved in processes as diverse as cell adhesion, apoptosis, the cell cycle, enzyme regulation, responses to stimuli and immunity [26]. Quantitative analysis of aMMP-8 in saliva and oral rinse samples has been performed previously, and higher levels were reported in people with periodontitis [27]. It is known that MMP-8 is mainly produced by polymorphonuclear leukocytes (PMNs) [28], but periodontal ligament fibroblasts and epithelial cells could also produce this protein. MMP-8 can promote the degradation of connective tissue extracellular matrix and accelerate the development of lesions. During inflammation, the quantity of PMNs in saliva increases, and proinflammatory cytokines in the gingival groove stimulate the further release inflammatory cytokines such as TNF-α, IL-6 via autocrine and paracrine pathways. As a result, the area of inflammation becomes enlarged, PMNs are further activated, and aMMP-8 is produced. PMNs and aMMP-8 permeating from the gingival groove eventually enter the saliva. As reported in some previous studies, tissue inhibitor of matrix metalloprotease-1 (TIMP-1) levels was screened in oral rinse samples by Amersham ELISA and a test utilizing a chromogenic substrate, and it was found that MMP-8/TIMP-1 M ratios increased together with periodontal inflammatory burden when measured by dentoELISA [17]. Although we mainly focused on aMMP-8 in the present study, we believe that further investigations of aMMP-8 jointly with its inhibitor TIMP-1 are still needed in our future studies.

There were no significant differences in aMMP-8 levels in oral rinse samples from different groups, which was an unexpected result of the present study. The results of correlation analysis indicated that there was no significant statistical association between the two biological fluids. As we know, saliva was the main fluid in oral rinse samples, and it can also be regarded as a diagnostic fluid. But unlike GCF, it reflects the oral status more generally and more unspecificly. The source of any oral biomarker should always be taken into account when analysing its function, and MMP-8 can possibly originate from a number of different sources including GCF, and salivary glands, as well as secretion from buccal mucosa. Further exploration of the exact contribution of these potential sources to oral MMP-8 levels would be interesting but also extremely challenging.

Nevertheless, we did observe statistical significance of the MMP-8 levels in GCF samples from the healthy and diseased participants. A lack of consistency in oral rinse samples from patients with chronic periodontitis has been largely attributed to the complexity and difficulty in determining disease status using the currently available diagnostic methods [29–31]. Various molecules presented in the saliva of periodontitis patients may be associated with chronic periodontitis, including enzymes such as collagenases, acute-phase proteins, and markers of bone loss [32, 33]. Quantitative analysis of aMMP-8 in the complex saliva samples is therefore difficult and requires further investigation.

The DentoELISA aMMP-8 immune detection system can be used to measure the aMMP-8 levels in point-of-care GCF and oral rinse samples within 15 min of collection in the clinic. DentoELISA can also be used to quantify aMMP-8 levels in plasma or other clinical samples. Our results clearly demonstrate the usefulness of dentoELISA for quantitative determination of aMMP-8, and indicate that this protein may be a useful biomarker for diagnosis of periodontitis.

Some limitations need to be addressed when extrapolating our current results for further research. First, this is a cross-sectional study without longitudinal observation, hence more thorough investigation with longitudinal design are still needed to shed more light on the progression of periodontitis. Second, since the imbalance between MMP-8 and its inhibitor TIMP-1 is considered to initiate periodontal disease [8], we think that it would be much valuable to perform further analyses on both MMP-8 and TIMP-1 in the future. Moreover, it is expected that further studies with larger sample size and more comprehensive study design will reveal more of this issue and yield better explanation of the intrinsic mechanism of the function of MMP-8 in periodontitis.

## Conclusion

Measurement of aMMP-8 levels in GCF has potentiality for complementary early diagnosis of chronic periodontitis disease and its associated inflammation in the Chinese population.

## Abbreviations

AL: Attachment loss
aMMP-8: Active matrix metalloproteinase-8
BOP: Bleeding on probing
GCF: Gingival crevicular fluid
MMP-8: Matrix metalloproteinase-8
PD: Pocket probing depth
